# Integration of FTIR Spectroscopy and Machine Learning for Kidney Allograft Rejection: A Complementary Diagnostic Tool

**DOI:** 10.3390/jcm14030846

**Published:** 2025-01-27

**Authors:** Luís Ramalhete, Rúben Araújo, Miguel Bigotte Vieira, Emanuel Vigia, Inês Aires, Aníbal Ferreira, Cecília R. C. Calado

**Affiliations:** 1 Blood and Transplantation Center of Lisbon, Instituto Português do Sangue e da Transplantação, Alameda das Linhas de Torres, No. 117, 1769-001 Lisbon, Portugal; 2 NOVA Medical School, Universidade NOVA de Lisboa, 1169-056 Lisbon, Portugal; rubenalexandredinisaraujo@gmail.com (R.A.);; 3 iNOVA4Health—Advancing Precision Medicine, RG11: Reno-Vascular Diseases Group, NOVA Medical School, Faculdade de Ciências Médicas, Universidade NOVA de Lisboa, 1169-056 Lisbon, Portugal; 4 Nephrology Department, Hospital Curry Cabral, Unidade Local de Saúde São José, 1049-001 Lisbon, Portugal; 5 Centro Hospitalar Universitário de Lisboa Central, Hepatobiliopancreatic and Transplantation Center—Curry Cabral Hospital, 1069-166 Lisbon, Portugal; 6 ISEL—Instituto Superior de Engenharia de Lisboa, Instituto Politécnico de Lisboa, R. Conselheiro Emídio Navarro 1, 1959-007 Lisbon, Portugal; cecilia.calado@isel.pt; 7 Institute for Bioengineering and Biosciences (iBB), The Associate Laboratory Institute for Health and Bioeconomy–i4HB, Instituto Superior Técnico (IST), Universidade de Lisboa (UL), Av. Rovisco Pais, 1049-001 Lisbon, Portugal

**Keywords:** kidney allograft, rejection, biomarkers, machine learning, FTIR spectroscopy

## Abstract

**Background**: Kidney transplantation is a life-saving treatment for end-stage kidney disease, but allograft rejection remains a critical challenge, requiring accurate and timely diagnosis. The study aims to evaluate the integration of Fourier Transform Infrared (FTIR) spectroscopy and machine learning algorithms as a minimally invasive method to detect kidney allograft rejection and differentiate between T Cell-Mediated Rejection (TCMR) and Antibody-Mediated Rejection (AMR). Additionally, the goal is to discriminate these rejection types aiming to develop a reliable decision-making support tool. **Methods**: This retrospective study included 41 kidney transplant recipients and analyzed 81 serum samples matched to corresponding allograft biopsies. FTIR spectroscopy was applied to pre-biopsy serum samples, and Naïve Bayes classification models were developed to distinguish rejection from non-rejection and classify rejection types. Data preprocessing involved, e.g., atmospheric compensation, second derivative, and feature selection using Fast Correlation-Based Filter for spectral regions 600–1900 cm^−1^ and 2800–3400 cm^−1^. Model performance was assessed via area under the receiver operating characteristic curve (AUC-ROC), sensitivity, specificity, and accuracy. **Results**: The Naïve Bayes model achieved an AUC-ROC of 0.945 in classifying rejection versus non-rejection and AUC-ROC of 0.989 in distinguishing TCMR from AMR. Feature selection significantly improved model performance, identifying key spectral wavenumbers associated with rejection mechanisms. This approach demonstrated high sensitivity and specificity for both classification tasks. **Conclusions**: The integration of FTIR spectroscopy with machine learning may provide a promising, minimally invasive method for early detection and precise classification of kidney allograft rejection. Further validation in larger, more diverse populations is needed to confirm these findings’ reliability.

## 1. Introduction

A kidney transplant (KT) is a life-saving procedure that increases both life span and quality of life for individuals with end-stage kidney disease, as the transplant of a healthy kidney from a donor enables transplanted patients to regain kidney function while circumventing the burdens of dialysis [1,2,3]. However, and despite a successful kidney transplant, the final outcome depends on a delicate balance between avoiding allograft rejection and controlling or limiting the risks associated with immunosuppressants drugs therapeutics [4].

Kidney allograft rejection (KAR) can occur due to various mechanisms. Immunological factors play a significant role, such as the recipient’s immune system recognizing the transplanted kidney as foreign due to differences in human leukocyte antigens (HLA) mismatches and the presence of pre-formed or *de novo* anti-HLA or non-HLA antibodies. Additionally, non-adherence to immunosuppressive medication and infections, which can stimulate and trigger an immune response, also contribute to rejection [5,6,7].

The immunological mechanisms leading to KAR can be divided into two major types: T Cell-Mediated Rejection (TCMR) and Antibody-Mediated Rejection (AMR).

TCMR is a type of immune-mediated response, which involves the infiltration of T lymphocytes and macrophages, into the transplanted kidney tissue, leading to inflammation and prospective damage to the organ [8]. Notwithstanding, TCMR can occur at any time post-transplant, despite typically presenting itself within the first few months. This rejection process involves the following steps: immune system activation based on the degree of HLA incompatibilities between donor and recipient; immune cell infiltration, where activated T lymphocytes migrate to the transplanted kidney, attracted by chemokines and cytokines released at the site of inflammation; release of inflammatory mediators, where infiltrated T lymphocytes release various inflammatory mediators, such as cytokines (e.g., interleukin-2, interferon-gamma), which promote further inflammation and recruit more immune cells to the site of rejection; and finally, allograft cellular damage [9,10,11,12].

Antibody-mediated rejection involves the production of antibodies by the recipient’s immune system against the HLA antigens present on the kidney allograft. These antibodies can bind to the kidney tissue and trigger an inflammatory response, leading to graft damage [10,13,14,15]. The timeline for AMR can vary depending on various factors, including individual patient characteristics and specific circumstances surrounding the transplant. It occurs when pre-existing antibodies are present, such as when patients have been exposed to foreign antigens through blood transfusions, previous transplants, or pregnancies. AMR can also occur in a more advanced stage whenever newly formed donor-specific antibodies (DSA) develop *de novo* [16,17,18].

The diagnosis of KAR has profound and far-reaching implications for patient outcomes. If left unchecked and/or untreated, rejection can initiate a cascade of detrimental effects, starting with graft dysfunction and potentially culminating in complete transplant failure. This not only compromises the patient’s health and quality of life but also necessitates the pursuit of alternative treatments, such as re-transplantation or long-term dialysis, which come with their own set of challenges and risks. Therefore, early and accurate diagnosis of rejection allows clinicians to implement timely and appropriate treatment strategies to combat rejection, essential to prevent or slow rejection [5,19].

In order to detect allograft rejection, a variety of techniques can be used, such as clinical evaluation, allograft biopsy, histopathology, molecular assays, imaging modalities, and laboratory tests. Together, these methods support the accurate assessment of allograft rejection and the direction of clinical interventions. Clinical assessment offers initial indications of allograft rejection. However, allograft biopsy, considered the gold standard, provides a more detailed evaluation of the rejection process. It enables histopathologists to assess critical aspects such as interstitial inflammation, tubulitis, endothelialitis, and fibrosis. Additionally, it identifies specific cell types, including lymphocytes and macrophages, while evaluating the degree and patterns of immune cell infiltration [19]. Although biopsies are critical, they involve invasive procedures with associated risks, such as bleeding and kidney damage. Additionally, biopsies are prone to sampling errors, which can lead to incorrect conclusions and potentially irreversible consequences for patients. This underscores the urgent need for biomarkers of allograft rejection that rely on non- or minimally invasive techniques.

However, it is important to emphasize that the proposed FTIR spectroscopy and machine learning approach is not intended to replace renal biopsy, which remains the gold standard for diagnosing kidney allograft rejection. Instead, this method is best positioned as a complementary tool that can provide clinicians with valuable information for stratifying the risk of rejection events. The integration of FTIR spectroscopy with machine learning allows for a non-invasive, high-throughput evaluation of patients, which could be particularly useful in monitoring patients over time (near-real time longitudinal studies) and identifying those at a higher risk of rejection. This underscores the urgent need for biomarkers of allograft rejection that rely on non- or minimally invasive techniques. In this context, diverse studies have explored approaches such as urinary and blood omics, including transcriptomics, cell-free DNA (cfDNA) analysis, proteomics, and others [20,21,22,23,24,25,26,27,28]. However, most of these studies presents critical constraints, e.g., not including the immune status or the mechanisms of rejection as based on biopsy analysis, being reliant on a low diversity of etiological diseases leading to allograft dysfunction [20]. Furthermore, conventional omics include complex techniques difficult to implement in large-scale populations.

An alternative technique, enabling to acquire in high sensitivity and specificity the system metabolic status, is based on FTIR spectroscopy [29], which has been applied to biofluids analysis to capture the patient’s physiological status [30,31,32], genotoxic effects [33], and medical diagnosis [34,35]. In allograft rejection, it is worthy to highlight the FTIR spectroscopic analyses of lymphocytes isolated from peripheral blood, enabling to discriminate B and T-lymphocytes with 89% accuracy [31], and lymphocytes activation [36]. Despite these promising results, the analysis requires a preliminary isolation step of these lymphocyte populations. Also, lymphocyte activation is not necessarily correlated with immune rejection processes. More recently, a study based on serum analysis, validated with 28 allograft biopsies, from 21 kidney-transplanted patients, enabled to discriminate patients without rejection processes and cellular rejection processes (AUC = 0.98) [37].

The present work aims to evaluate the serum FTIR spectroscopic analysis to detect the rejection process and to discriminate the TCMR from the AMR processes, as based on a rapid, economic, and high-throughput analysis, based on the spectra acquisition of a simple drop of serum (i.e., of 25 µL) after a simple dehydration step, and using a microplate with 96 wells for the spectra acquisition.

## 2. Materials and Methods

### 2.1. Study Population

This retrospective study included 41 adult kidney transplant recipients from living or deceased donors. Multiple biopsies were performed for some individuals at different time points, yielding 81 biopsies in total. Among these patients, eight demonstrated sequential changes in rejection status: three progressed from no rejection to TCMR, four from no rejection to AMR, and one transitioned from no rejection to TCMR, followed by AMR. Additionally, four patients had multiple biopsies revealing both TCMR and AMR at various time points. These details underscore the complexity of the cohort, which was accounted for during data analysis. The study was approved by the ULS São José Ethics Committee (approval numbers 454/2017 and 1215/2022), and all patients provided informed consent.

Patients were selected to minimize the fact that, apart from the target variable (rejection), all other demographic and major clinical variables were not statistically different among patient groups, as described in [38] (Table 1 and Table 2). A total of 81 serum samples were considered, that are matched to corresponding kidney biopsies, performed either for non-protocol or protocol indications. Biopsy results classified 56 samples as rejection cases, comprising 12 TCMR and 44 AMR samples. The remaining 25 samples were classified as non-rejection. Table 1 and Table 2 present the demographic and clinical data, along with the corresponding statistical analyses comparing two populations. Analysis 1 evaluates rejection versus non-rejection, while Analysis 2 compares humoral rejection and cellular rejection. Except DSAs, in rejection versus no rejection, no statistically significant differences (*p* > 0.05) were observed between the study populations in terms of age, sex, donor type (living or deceased), HLA mismatches, serum creatinine, serum urea, or whether the transplanted organ was a kidney alone or a simultaneous kidney–pancreas (Table 1 and Table 2).

### 2.2. FTIR Spectra Acquisition

Twenty-five μL of serum diluted at 1/10 in Milli-Q^®^ water were plated into a 96-well Si microplate and then dehydrated in a desiccator for 150 min under vacuum. Spectra were collected using a FTIR spectrometer (Vertex 70, Bruker, Germany), equipped with an HTS-XT (Bruker, Germany) accessory. Each spectrum represented 64 coadded scans, acquired in transmission mode between 400 and 4000 cm^−1^, with a resolution of 2 cm^−1^. The first well of the 96-well microplate was left without a sample and the corresponding spectra was used as background, according to the HTS-XT manufacturer.

### 2.3. Data Analysis

The demographic and clinical variables of the different populations in this study (Table 1 and Table 2) were statistically evaluated to assess the potential effect of confounding factors on the data set [38]. Appropriate tests, including the Chi–square test and Mann–Whitney U, were performed using GraphPad Prism version 8.0.2 for Microsoft Windows (GraphPad Software, San Diego, CA, USA).

Serum spectra were pre-processed by atmospheric compensation and with baseline correction (Rubber-Band) or in alternative with second derivative based on a Savitzky–Golay filter, with a second polynomial and a corresponding window size of 17 points.

The spectra *t*-distributed stochastic neighbor embedding method (*t*-SNE) was conducted. The *t*-SNE is a non-linear dimensionality reduction technique that maps high-dimensional data into a lower-dimensional space, typically two or three dimensions, while preserving the relative distances between data points. By maintaining local relationships and proximities, *t*-SNE effectively highlights patterns and structures within complex data sets, such as clusters or groupings, which might otherwise be obscured in higher-dimensional spaces. This method has gained popularity in fields such as bioinformatics and single-cell analysis due to its ability to uncover meaningful insights from intricate data distributions [39,40].

Predicting Naïve Bayes models were developed. This model is a probability classification algorithm that uses the Bayes theorem to make predictions about the likelihood that a certain event will occur given the input data. It assumes that each feature of the input data is independent of every other feature. Despite this presumption, Naïve Bayes has seen widespread application and has demonstrated strong performance in a variety of real-world applications, including healthcare [37,41]. By multiplying the probabilities of each feature in a given class, it calculates the likelihood that a data point will belong to that class, selecting the class with the highest likelihood as the prediction [42,43]. Leave-One-Out Cross-Validation (LOOCV) was used to estimate the performance of the predicting models. The classification model’s overall performance was quantified by area under the receiver operating characteristic (AUC), sensitivity, specificity, accuracy, precision, and F-1 score.

To identify relevant features based on their correlation with the target variable and the amount of information they convey, feature selection was performed using Fast Correlation-Based Filter (FCBF). The FCBF, an entropy-driven method, prioritizes spectral features by evaluating their significance in distinguishing target groups (e.g., rejection vs non-rejection). This algorithm assigns scores ranging from 0 to 1, with higher values indicating greater importance for group differentiation [44].

Spectra pre-processing by atmospheric correction was conducted with OPUS^®^ software, version 6.5 (Bruker, Germany). The remaining spectra pre-processing and processing (t-SNE, feature selection, and Naïve Bayes models) were conducted with Orange 3 version 3.35.0 (Bioinformatics Lab, University of Ljubljana, Ljubljana, Slovenia).

## 3. Results

To effectively address the challenge of identifying and categorizing rejection states, a two-step analytical approach was implemented. First, it was evaluated the discrimination between rejection from non-rejection. This foundational step ensures robust identification of rejection cases, providing a reliable starting point for subsequent analysis. In the second phase, the focus shifted to differentiating, on the rejection cases, between the two rejection mechanisms—TCMR and AMR—enhancing the precision of this evaluation. Therefore, the following analyses using serum FTIR spectra were conducted:

Analysis 1: To distinguish patients experiencing rejection from those without rejection.

Analysis 2: To discriminate between TCMR and AMR, focusing exclusively on these two rejection mechanisms for detailed classification.

### 3.1. Rejection Versus Non-Rejection

**Figure 1:**
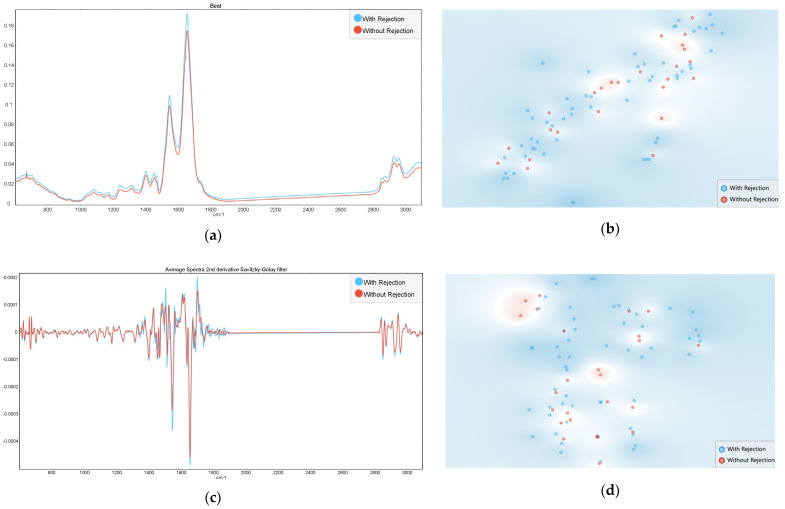
Average serum spectra (**a**,**c**) and corresponding *t*-SNE (**b**,**d**) from samples associated with rejection processes (in blue) and non-rejection processes (in red), considering the following spectra pre-processing: (**a**,**b**) Baseline correction, (**c**,**d**) second derivative spectra, with the amide region highlighted.

It was considered spectra from 25 serum samples from patients without rejection processes, and 56 serum samples from patients with rejection processes, including TCMR (*n* = 12) and AMR (*n* = 44). It was evaluated spectra with baseline correction or in alternative the second derivative spectra, since derivative resolves superimposed bands, theoretically increasing the spectra information. In relation to the second derivative spectra, it was considered the sub-regions between 600 to 1900 and between 2800 and 3400 cm^−1^, to avoid regions with a low signal to noise ratio. As expected, the average serum spectra were highly similar between the two patient groups, reflecting the predominant biomolecules present in serum (Figure 1). This overall similarity posed challenges for *t*-SNE, which was unable to highlight distinct patterns according to the patient groups (i.e., with and without rejection) (Figure 1).

**Figure 2:**
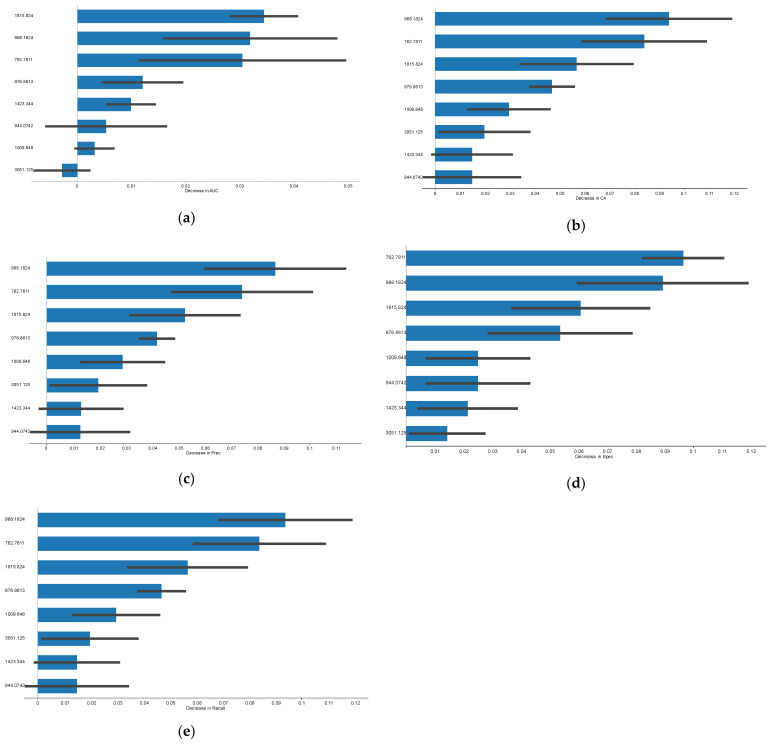
Feature importance plots in the Naïve Bayes predicting model of rejection. Features contribution for: (**a**) AUC, (**b**) Accuracy, (**c**) Precision, (**d**) Specificity, (**e**) Sensitivity. The black bars correspond to a 95% confidence interval.

Naïve Bayes prediction models were developed based on the second derivative spectra, that, however, resulted in a low performance with an AUC of 0.59. These findings underscore the constraints of the current models, emphasizing the need for further refinement or alternative approaches to achieve better performance [36,45]. To improve the model’s performance, a feature selection based on FCBF was implemented. Eight spectral bands were selected according to these bands’ impact on the AUC and model’s performance (Figure 2). These features plots provide a visual representation of the relative importance of the most relevant features, helping to identify the key variables that significantly contribute to the model’s predictive performance, thereby aiding in feature selection and interpretation of results. From the feature importance plots (Figure 2), it can be observed that certain bands exhibit higher importance values, indicating a stronger influence on the model’s predictions. Overall, the wavenumber ~763 cm^−1^ and ~968 cm^−1^ were the most influential in the model performance. Other bands with relevance, despite with lower impact than the previous ones, include ~968 cm^−1^ and ~1815 cm^−1^.

**Figure 3:**
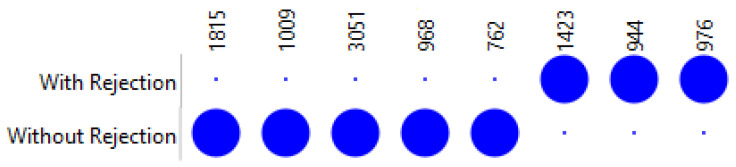
Dot Matrix Plot, from the eight most informative spectral bands selected by FCBF to discriminate rejection from non-rejection serum samples.

From these eight spectral bands, three presents higher values on the rejection group, at 1423, 944, and 976 cm^−1^, while the other five presented higher values on the non-rejection group, at 762, 968, 1009, 1815, and 2051 cm^−1^ (Figure 3).

Naïve Bayes models based on these eight bands, presented a significant improved performance, pointing an AUC of 0.945 (Table 3). These results validate the effectiveness of feature selection in enhancing classification performance.

### 3.2. Humoral Versus Cellular Rejection

**Figure 4:**
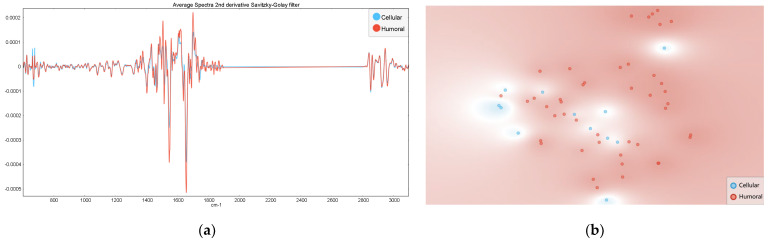
Average serum second derivative spectra (**a**) and the corresponding *t*-SNE (**b**) for humoral rejection samples (in red), for cellular rejection (in blue), and non-rejection samples (in green).

For samples classified as humoral (n = 44) or cellular rejection (n = 12), several noticeable differences were observed in the averaged second derivative spectra, that however did not result in clusters in the *t*-SNE plot according to the rejection mechanism (Figure 4). The prediction Naïve Bayes model based on the second derivative spectra also presented a low performance with an AUC of 0.68.

**Figure 5:**
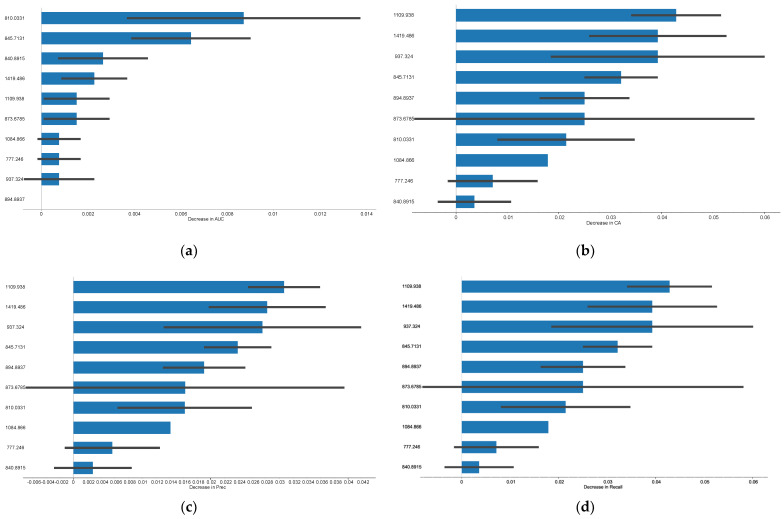
Feature importance plots in the Naïve Bayes model to predict the mechanism of rejection. Features contribution for: (**a**) AUC e, (**b**) Accuracy, (**c**) Precision, (**d**) Sensitivity. The black bars correspond to a 95% confidence interval.

As previously, in order to improve the model’s performance, spectral bands were selected based on FCBF, that lead to improve the model’s performance as pointed in Figure 5. This figure highlights feature importance, identifying key variables in the data set and aiding in the interpretation of the Naïve Bayes model’s results. Certain variables, such as wavenumbers ~1110 cm^−1^ and ~1420 cm^−1^, exhibit higher importance, reflecting their stronger influence on the model’s predictions.

**Figure 6:**
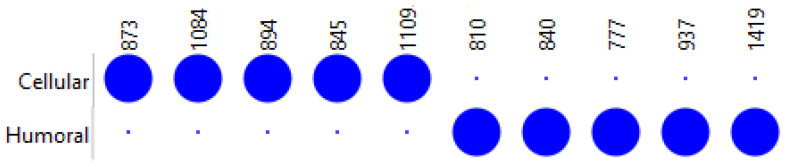
Dot Matrix Plot from the 10 most informative wavenumber selected by FCBF to discriminate non-rejection from humoral or cellular rejection serum samples.

It was possible to identify 10 wavenumbers that may prove useful for discriminating between humoral rejection, and cellular rejection, as observed in Figure 6. In this case, five wavenumbers (~874, ~1085, ~895, ~846, and ~1110 cm^−1^) were more prominent in cellular rejection, and five wavenumbers (~810, ~841, ~777, ~937, and ~1420 cm^−1^) in non-rejection in humoral rejection.

The Naïve Bayes model based on the 10 spectral bands to predict the rejection mechanism presented an excellent performance with an AUC of 0.989 (Table 4). Overall, the model misclassified two samples that were unequivocally humoral rejection, incorrectly categorizing them as cellular rejection when analyzed using the Naïve Bayes algorithm. Additionally, the data set included seven samples classified as mixed rejection, which, although exhibiting characteristics of both cellular and humoral rejection, were predominantly humoral in nature. These mixed rejection samples may have added complexity to the classification process, potentially influencing the model’s performance.

## 4. Discussion

As previously stated, several methods and tests are currently available to detect rejection in kidney allografts. Blood tests measure levels of specific proteins, enzymes, or markers such as creatinine, blood urea nitrogen, and cystatin C [46,47]. Urine tests can detect signs of inflammation, such as white blood cells or red blood cells, or allograft damage, such as proteinuria. Medical imaging techniques, including ultrasound, computed tomography, and magnetic resonance imaging, provide information on potential rejection indicators, such as swelling or inflammation of the allograft. The gold standard remains allograft biopsy, which, despite its invasive nature, offers precise insights into the health and function of the transplanted kidney.

The optimal method for detecting rejection may vary depending on the patient and the severity of rejection, often requiring a combination of approaches. For instance, high-risk patients may be monitored regularly with blood and urine tests, followed by a biopsy if signs of rejection arise. In such cases, a protocol biopsy scheme may be implemented. Conversely, low-risk patients may only require periodic monitoring with blood and urine tests.

Despite their utility, many biomarkers lack adequate sensitivity and specificity for detecting rejection. For example, proteinuria > 1.0 g/24 h, detected up to 3 months post-transplant, showed an AUC of 0.64 for graft failure with 85% specificity and 48% sensitivity [48]. To address these limitations, new techniques have emerged, including cfDNA and the Molecular Microscope Diagnostic System (MMDx) [27,49,50]. cfDNA, specifically donor-derived cfDNA (dd-cfDNA), is released into the bloodstream when a transplanted organ is damaged during rejection. MMDx uses DNA sequencing and machine learning to identify gene expression patterns in allograft biopsies associated with rejection, enabling earlier diagnosis. These methods offer advantages, such as the minimally invasive nature of dd-cfDNA testing and its ability to detect rejection earlier. However, challenges remain, including limited availability, high costs, and the need for further validation of their accuracy and reliability (Table 5).

Kidney transplant patients have varied characteristics, including differences in age, genetics, comorbidities, and treatment regimens, which can influence allograft outcomes. Additionally, factors such as donor type (living or deceased), organ preservation methods, and time since transplantation may affect the rejection process.

Therefore, to ensure that the presented models in the current study have broad applicability and reliability in the real-world clinical environment, it is essential to further validate and test across diverse patient populations. This expanded validation should include larger data sets that encapsulate a wider range of patient demographics and clinical scenarios. Additionally, multicentric studies involving different transplantation centers would provide a more comprehensive understanding of the models’ performance in various clinical settings. This study was designed as an exploratory proof of concept to assess FTIR spectroscopy as a minimally invasive, high-throughput auxiliary diagnostic tool for kidney allograft rejection. For instance, transcriptomic analyses could identify changes in gene expression associated with rejection pathways, such as those involving interferon gamma signaling or complement activation [58,59]. These findings could help link specific spectral features to molecular events that occur during rejection. Similarly, proteomic studies might reveal unique protein signatures in the serum that are indicative of rejection, providing complementary biomarkers to the spectral data. Metabolomic profiling could further enhance these findings by identifying small molecules and metabolic changes linked to graft injury or immune activation [20].

Also, the inclusion of diverse patient data would contribute to refining the models, making them more robust against overfitting to specific patient groups or clinical conditions. This will enhance the models’ utility in accurately predicting KAR, thereby supporting clinicians in making informed decisions and potentially improving patient outcomes. Furthermore, continuous monitoring of the model’s performance in real-time clinical settings post-implementation will provide insights into its long-term efficacy and areas for improvement.

When comparing the performance of more conventional assays (Table 5), such as cystatin C, with AUCs of 0.64 [46], or even these more advanced and expensive modern solutions like MMDx, with AUCs of 0.87 [55], and dd-cfDNA, with AUCs > 0.89 [52], to the performance obtained in the first model for rejection vs. no-rejection serum samples, the Naïve Bayes model achieved an AUC-ROC of 0.945.

In this study, the identification of specific wavenumbers in the FTIR spectra provides crucial molecular insights into the biochemical changes associated with kidney transplant rejection. Each wavenumber represents distinct molecular vibrations, highlighting key metabolic and structural alterations in serum components. The observed biochemical changes in KAR can be further elucidated by examining the specific wavenumbers identified in the FTIR spectra, which highlight distinct molecular interactions. Collectively, these spectral insights provide a robust framework for understanding the molecular underpinnings of kidney transplant rejection and emphasize the potential of FTIR spectroscopy as a non-invasive diagnostic tool.

Regardless of these findings, certain considerations must be acknowledged when comparing the performance of the the Naïve Bayes model in this study, (AUC-ROC: 0.945), to other diagnostic assays such as cystatin C, MMDx, and dd-cfDNA [46,55]. First, these comparisons should account for the data set’s size and composition. The relatively limited data set in this study may affect the generalizability of the findings, whereas established assays like cystatin C, MMDx, and dd-cfDNA are often evaluated using larger, more diverse data sets, providing a more robust representation of their clinical effectiveness [46].

Additionally, the fundamental differences in methodology between the ML models and traditional assays must be considered. While this study’s models rely on algorithmic interpretations of FTIR spectroscopy data, traditional assays often utilize distinct biological markers or detection methods. These methodological differences complicate direct comparisons, as each approach has unique strengths and limitations.

The results presented here are promising and suggest potential clinical utility; however, they must be interpreted with these contextual nuances in mind. Further research and validation with larger, more diverse data sets is essential to fully assess the effectiveness and applicability of these models relative to established assays.

For discrimination between humoral rejection, and cellular rejection, the Naïve Bayes model outperformed established methods (e.g., MMDx, dd-cfDNA) with an AUC of 0.989 and consistently high specificity. The model correctly identified all cellular rejection cases (12 of 12) and achieved accuracies of 96%.

By incorporating this model into standard protocols, healthcare providers could enhance early detection of rejection episodes, improving patient outcomes and prolonging graft survival. To translate FTIR spectroscopy and machine learning into clinical practice, it is essential to establish clear protocols for sample collection, processing, and analysis. Standardized guidelines would ensure the reproducibility of results and facilitate the implementation of this technique in routine diagnostic workflows. FTIR spectroscopy into the diagnostic process for kidney transplant rejection offers a promising avenue for enhancing patient outcomes while potentially reducing healthcare costs. FTIR spectroscopy enables the rapid and cost-effective analysis of serum samples, facilitating the early detection of cellular rejection processes. A study demonstrated that FTIR spectroscopy, combined with machine learning algorithms, could predict cellular rejection with high sensitivity and specificity (AUC > 0.984), suggesting its suitability for routine analyses and large-scale studies [37].

Early identification of rejection episodes through FTIR spectroscopy may reduce the reliance on invasive biopsy procedures, which are associated with risks such as bleeding, infection, and increased healthcare expenditures [60,61,62]. Moreover, managing complications like AMR significantly escalates healthcare costs. For instance, the mean healthcare costs attributable to AMR in kidney transplant recipients are four times higher than those of matched controls, amounting to an additional $13,066 per patient in the 60 days prior to AMR diagnosis and $35,740 per patient per year in the 2 years following the diagnosis [63], with treatment costs for episodes in the United States ranging between $49,000 and $155,000 per episode [64]. Additionally a 12-month protocol biopsy for subclinical rejection screening in KT cost $13,318 per quality-adjusted life year (QALY) gained, while biomarker-based screening was cost-effective only if the biomarker cost was below $700 per test [65].

By facilitating early detection and intervention, FTIR spectroscopy has the potential to mitigate such costly complications, thereby improving patient outcomes and reducing the financial burden on healthcare systems. However, comprehensive cost-effectiveness analyses are necessary to fully evaluate the economic benefits of implementing FTIR spectroscopy in clinical practice. Future studies should focus on comparing the costs and outcomes associated with FTIR-based diagnostics versus traditional methods to determine its viability as a standard tool for monitoring kidney transplant patients.

Additionally, a comprehensive cost-effectiveness analysis should be conducted to evaluate the feasibility of adopting FTIR spectroscopy compared to existing diagnostic modalities. Such an analysis would consider not only the costs of instrumentation and implementation but also the potential savings from reducing invasive procedures, such as biopsies, and improving patient outcomes through earlier intervention. These efforts are critical to bridge the gap between experimental findings and real-world clinical applications. However, despite its strong performance, the Stacking model requires further validation and rigorous testing across diverse patient populations and clinical settings to ensure its generalizability and reliability. Continued research and refinement are critical to fully realize its potential and maximize its impact on transplant medicine.

## 5. Study Limitations and Directions for Future Research

While this study demonstrates the potential of integrating FTIR spectroscopy with machine learning for detecting and classifying KAR, several limitations should be acknowledged. Addressing these limitations will be crucial for the broader application and validation of this approach.

The primary limitation of this study is the relatively small data set, which may not fully capture the diversity of clinical scenarios encountered in kidney transplantation. Factors such as patient demographics, donor characteristics, and transplantation conditions can significantly influence rejection processes. Although robust statistical techniques, including LOOCV, were employed to enhance the reliability of results, the restricted sample size remains a constraint. Future multicenter studies should focus on expanding data sets to include a broader range of patient demographics and clinical scenarios. This will ensure the generalizability of findings and support the development of more robust predictive models [27,49].

Another challenge was the imbalance in sample sizes between rejection groups, reflecting the natural prevalence of AMR over TCMR in the studied population. While machine learning models accounted for this imbalance, future research should aim to achieve a more balanced group distribution to improve statistical power and the accuracy of classification models. This could involve targeted recruitment of cases with less frequent rejection types or developing techniques to handle imbalanced data sets more effectively [66,67,68]. Additionally, while FTIR spectroscopy offers a minimally invasive and high-throughput alternative, its integration with molecular analyses could further validate the biological mechanisms underlying the spectral signatures identified in this study. Techniques such as transcriptomics, proteomics, and metabolomics hold promise for uncovering the biochemical pathways associated with rejection processes. By combining these molecular approaches with FTIR spectroscopy, future research could establish a comprehensive diagnostic framework linking molecular-level insights with spectral patterns. This integrated framework could validate FTIR-based findings and facilitate the discovery of novel biomarkers for early detection, classification, and risk stratification of rejection episodes [69,70].

Lastly, larger and more diverse studies are necessary to confirm the utility of FTIR spectroscopy across varied clinical settings. Multicenter collaborations would provide the opportunity to test the models in real-world conditions and refine their performance. Such efforts would also allow the comparison of FTIR-based diagnostics with established methods, such as cfDNA and the MMDx, to contextualize its clinical utility further [27,49]. By addressing these limitations and exploring the proposed directions for future research, this approach has the potential to enhance diagnostic precision, reduce reliance on invasive biopsies, and support more personalized management strategies for kidney transplant patients.

## 6. Conclusions

Kidney transplantation remains a critical treatment option for end-stage kidney disease, yet managing allograft rejection poses ongoing challenges. This study evaluated the potential of integrating FTIR spectroscopy with machine learning algorithms to enhance the detection and classification of KAR. The findings demonstrated that machine learning models, particularly Naïve Bayes, effectively distinguished between rejection and non-rejection cases and showed promise in differentiating TCMR from AMR. This differentiation is vital, as it directly informs therapeutic strategies and transplant recipient management. The results suggest that combining FTIR spectroscopy with advanced machine learning techniques offers a minimally invasive approach for early rejection detection and precise identification of rejection mechanisms. In conclusion, the integration of FTIR spectroscopy with machine learning holds significant potential for improving the diagnosis and management of KAR. By enabling early detection and accurate differentiation between cellular and humoral mechanisms, this approach could facilitate more tailored and effective treatments, ultimately enhancing patient outcomes. Future research should focus on expanding data sets and validating these models across diverse patient populations to fully realize their clinical utility. While this method offers substantial promise, it is important to emphasize that it is not intended to replace renal biopsy, which remains the gold standard for definitive diagnosis. Instead, it can complement biopsy findings by supporting early detection, risk stratification, and guiding surveillance strategies, thereby contributing to a more comprehensive approach to managing kidney transplant patients.

## 7. Patents

In addition to the findings presented, it should be noted that certain aspects of this work are currently under a patent request, specifically under the filing provisional number 119289, reflecting the novel approach and potential applicability of this method in clinical settings.

## Figures and Tables

**Table 1:** Characteristics of patients not presenting a rejection process and patients presenting a rejection process, independently of the rejection mechanism, used to acquire 81 serum samples. The *p*-value from the statistical analysis comparing these groups is indicated.

Characteristics	Without Rejection (Samples n = 25) (Patients n = 20)	With Rejection (Samples n = 56) (Patients n = 27)	*p*-Value
	Median or % (SD)	Median or % (SD)	
Age (years)	45 (12.31)	43 (13.24)	0.308 *
Gender (% male)	74% -	68%	0.757 **
Gestations	0.16(0.4)	0.71 (1.6)	0.4521 *
Donor type (% from deceased donor)	90%	89%	1.00 **
Transplanted organ			
Kidney alone	65%	74%	0.233 **
Kidney plus pancreas	35%	26%	
Rejection classification grade (based on biopsy)			
Non-rejection	25		0.000 **
No alteration	13 (52%)		
Only pancreatic rejection	2 (8%)		
*de novo* membranous glomerulonephritis	2 (8%)		
Polyomavirus-associated nephropathy	2 (8%)		
*de novo* glomerulopathy	2 (8%)		
Primary non-function	1 (4%)		
Allograft nephritis	1 (4%)		
Calcineurin Inhibitor Toxicity	1 (4%)		
Human immunodeficiency virus-associated nephropathy	1 (4%)		
Cellular		12	
Borderline		1 (8%)	
IA		8 (67%)	
IB		1 (8%)	
IIA		1 (8%)	
III		1 (8%)	
Humoral		44	
Borderline		3 (7%)	
1		13 (30%)	
2		9 (20%)	
Chronic		12 (27%)	
Mixed predominant humoral		7 (16%)	
DSAs (% yes)	24%	52%	0.028 **
PRA CDC Maximum	13.8 (17.4)	15.7 (28.5)	0.450 *
HLA mismatches			0.134 *
HLA Class I (A and B)	2.74 (0.9)	2.48 (1.0)	
HLA Class II (DR)	1.4 (1.0)	1.03 (0.6)	
Serum creatinine	1.5 (2.1)	1.8 (2.0)	0.303 *
Blood urea nitrogen	66 (49)	60 (37)	0.881 *
Urea	812(43)	103 (68)	0.444 *

SD, Standard deviation; *, Mann–Whitney U; **, Chi–square; DSAs, Donor-Specific Antibodies; PRA CDC, panel-reactive antibody by complement-dependent cytotoxicity test.

**Table 2:** Characteristics of patients presenting cellular (TCMR) or humoral (AMRs) rejection processes, used to acquire 56 serum samples. The *p*-value from the statistical analysis comparing these groups is indicated.

Characteristics	With Rejection (n = 56)		*p*-Value
	Cellular (Samples n = 12) (Patients n = 9)	Humoral (Samples n = 44) (Patients n = 24)	
	Median or % (SD)	Median or % (SD)	
Age (years)	46.0 (13.2)	45.5 (13.5)	0.65 *
Gender (% male)	55%	73%	0.35 **
Gestations	0.89 (1.5)	0.64 (1.7)	0.493 *
Donor type (% from deceased donor)	89%	86%	1 **
Transplanted organ			
Kidney alone	77%	79%	1 **
Kidney plus pancreas	23%	21%	
Rejection classification grade at time of biopsy			
Cellular			
Borderline	1 (8%)		0.000 *
IA	8 (67%)		
IB	1 (8%)		
IIA	1 (8%)		
III	1 (8%)		
Humoral			
Borderline		3 (7%)	
1		13 (30%)	
2		9 (20%)	
Chronic		12 (27%)	
Mixed predominant humoral		7 (16%)	
DSAs (% yes)	42%	55%	0.63 **
PRA CDC Maximum	4.44 (7.3)	30.6 (33)	0.32 *
HLA mismatches			
HLA Class I (A and B)	2.4 (0.9)	2.5 (1.0)	0.73 *
HLA Class II (DR)	0.9 (0.8)	1.1 (0.6)	0.55 *
Serum creatinine	1.81 (1.2)	1.17 (2.2)	0.60 *
Blood urea nitrogen	57 (32)	44 (39)	0.45 *
Urea	78 (19)	74 (71)	0.908 *

SD, Standard deviation; *, Mann–Whitney U; ** Chi–square; DSAs, Donor-Specific Antibodies; PRA CDC, panel-reactive antibody by complement-dependent cytotoxicity test.

**Table 3:** Naïve Bayes algorithm models to discriminate rejection from non-rejection samples based on the second derivative spectra and of the eight most informative spectral bands.

	Model	AUC-ROC	Accuracy	F1-Score	Precision	Sensitivity	Specificity
600–1900 cm^−1^ and 2800–3400 cm^−1^	Naïve Bayes	0.587	0.543	0.554 * (0.507) ** (0.543) ***	0.675 * (0.380) ** (0.575) ***	0.541 * (0.760) ** (0.444) ***	0.663 * (0.446) ** (0.460) ***
eight most informative wavenumber	Naïve Bayes	0.945	0.926	0.927 * (0.885) ** (0.945) ***	0.929 * (0.852) ** (0.963) ***	0.926 * (0.920) ** (0.929) ***	0.923 * (0.929) ** (0.920) ***

AUC-ROC, Area under the ROC Curve; * average over classes; **, non-rejection; ***, rejection.

**Table 4:** Machine Learning algorithm models to discriminate non-rejection from humoral or cellular rejection serum samples based on the second derivative spectra of the 10 most informative wavenumbers and based on the wavenumber from 600 to 1900 cm^−1^ and 2800 to 3400 cm^−1^.

	Model	AUC-ROC	Classification Accuracy	F1-Score	Precision	Sensitivity	Specificity
600–1900 cm^−1^ and 2800 to 3400 cm^−1^	Naïve Bayes	0.678	0.518	0.545 * (0.449) ** (0.571) ***	0.808 * (0.297) ** (0.947) ***	0.518 * (0.917) ** (0.409) ***	0.808 * (0.409) ** (0.917) ***
10 most informative s wavenumber	Naïve Bayes	0.989	0.964	0.965 * (0.923) ** (0.977) ***	0.969 * (0.857) ** (1.000) ***	0.964 * (1.000) ** (0.955) ***	0.990 * (0.955) ** (1.000) ***

AUC-ROC, Area under the ROC Curve; * average over classes; ** results for cellular rejection; ***, results for humoral rejection.

**Table 5:** Overview of different methods for identify allograft rejection.

	No. of Patients	Cutoff	AUC	Sensitivity	Specificity	References
donor-derived Cell-free DNA	261	dd-cfDNA of ≥1%	--	0.24	0.95	[51]
	75	dd-cfDNA of ≥1%	0.89	0.80	0.80	[52]
	107	dd-cfDNA of ≥0.88%	0.64	0.38	0.85	[53]
	189	dd-cfDNA of ≥0.5%	0.83	0.73	0.73	[54]
Molecular Microscope^®^ diagnostic system	1745	--	0.87	0.85	--	[55]
	74	--	C statistic 0.81	--	--	[56]
	519	--	--	0.78	0.74	[57]

## Data Availability

The data presented in this study is available on request from the corresponding author.
